# MetaCarvel: linking assembly graph motifs to biological variants

**DOI:** 10.1186/s13059-019-1791-3

**Published:** 2019-08-26

**Authors:** Jay Ghurye, Todd Treangen, Marcus Fedarko, W. Judson Hervey, Mihai Pop

**Affiliations:** 1 Department of Computer Science, University of Maryland, College Park, MD USA; 2 Center for Bioinformatics and Computational Biology, University of Maryland, College Park, MD USA; 3 Department of Computer Science, Rice University, Houston, TX USA; 4 Center for Bio/Molecular Science & Engineering, United States Naval Research Laboratory, Washington, DC, USA

**Keywords:** Metagenomics, Variant detection, Scaffolding, Assembly

## Abstract

Reconstructing genomic segments from metagenomics data is a highly complex task. In addition to general challenges, such as repeats and sequencing errors, metagenomic assembly needs to tolerate the uneven depth of coverage among organisms in a community and differences between nearly identical strains. Previous methods have addressed these issues by smoothing genomic variants. We present a variant-aware metagenomic scaffolder called MetaCarvel, which combines new strategies for repeat detection with graph analytics for the discovery of variants. We show that MetaCarvel can accurately reconstruct genomic segments from complex microbial mixtures and correctly identify and characterize several classes of common genomic variants.

**Electronic supplementary material:**

The online version of this article (10.1186/s13059-019-1791-3) contains supplementary material, which is available to authorized users.

## Background

Sequencing of DNA directly extracted from microbial communities (metagenomics) has emerged as a key tool in the exploration of the role microbes play in human and environmental health. Large-scale studies enabled by metagenomic methods, such as MetaHIT [1] and the Human Microbiome Project (HMP) [2], have cataloged the complex microbial communities associated with the human body and have demonstrated their importance to human health. By eliminating the need for culturing, metagenomic sequencing has made it possible to explore a broader range of the microbes inhabiting our world and has led to the discovery of novel organisms and genes from complex samples [3–6].

Despite promising initial results, the reconstruction of the entire or even partial organisms from complex microbial mixtures remains a tremendous challenge. The assembly of metagenomic sequences is confounded by several factors: (i) uneven abundance of the different organisms found in a sample, (ii) genomic variation between closely related organisms, (iii) conserved genomic regions shared by distantly related genomes (inter-genomic repeats), and (iv) repetitive DNA within a single genome (intra-genomic repeats). All but the latter challenges are unique to metagenomic data and have not been the target of research until very recently.

Several genome assembly tools designed explicitly for metagenomic data have been developed in recent years. Among the most widely used are metaSPAdes [7] and MEGAHIT [8]; however, many other tools have been developed including MetaVelvet [9], IDBA-UD [10], Ray Meta [11], and Omega [12]. These tools effectively address the uneven coverage of metagenomic datasets, but virtually all of them “smooth out” small differences between co-occurring strains of organisms in order to enable the reconstruction of longer genomic segments from the mixture. Furthermore, the output of the assemblers is simply a collection of linear segments (contigs) that lacks the connection between the segments originating from the same organism. As a result, additional analyses are necessary to discover information about the adjacency of genomic segments (e.g., operon structure in bacteria), or large-scale genomic variants between co-occurring microbial strains. The latter information is of particular research interest in microbial ecology, for example, in the context of the lateral gene transfer [13] or understanding how genomic heterogeneity contributes to the stability of microbial communities [14].

The study of genomic variants in microbial communities is of considerable interest, and a number of computational tools have been developed to discover this information. The approaches are primarily based on read alignments to either complete genomes, as performed for example by metaSNV [15] and MIDAS [16], or against conserved genes, as performed by ConStrains [17] and StrainPhlan [18]. Strain variants can also be discovered directly from the output of the assembler, as done, for example, for diploid genomes through a colored de Bruijn graph approach [19], or in metagenomic data through the use of the SPQR tree data structure [20].

The discovery of genomic variants from assembly relies on the information contained in an assembly graph—a representation of the ambiguity in the reconstruction of the genome or metagenome. While many assemblers can output this information, an assembly graph can also be constructed post-assembly by linking together genomic contigs through the information provided by paired reads or other sources of information, using a computational process called scaffolding. While most existing genome and metagenome assemblers [7, 8, 10, 21] contain dedicated scaffolding modules [22], the output of these tools comprises linear paths that ignore the presence of genomic variants. An exception is stand-alone scaffolders such as Bambus 2 [23] or Marygold [20] that explicitly retain ambiguity in the assembly graph and use graph analyses to characterize specific genome variants.

Here we describe a new metagenomic scaffolding package called MetaCarvel, a tool that substantially improves upon the algorithms implemented in Bambus 2 and MaryGold. We show that MetaCarvel generates more contiguous and accurate scaffolds than one of the best performing stand-alone scaffolders, OPERA-LG [24], as shown by a recent study [25]. We also demonstrate that MetaCarvel is able to accurately detect a number of genomic variants, including regions with divergent sequence, insertion/deletion events, and interspersed repeats. MetaCarvel is released under the MIT open source license and is available at https://github.com/marbl/MetaCarvel.

## Results

Below we demonstrate and evaluate the performance of MetaCarvel by relying on a mixture of synthetic and real metagenomic datasets. We rely on mixtures of *Acinetobacter baumanii* strains sequenced as part of surveillance of a healthcare institution [26] to reveal the impact of heterogeneity on the quality of genome assemblies and to demonstrate that MetaCarvel can detect regions of high sequence divergence. The ability of MetaCarvel to detect insertion/deletion events is determined within a mixture of sequencing data derived from two *Escherichia coli* strains—organism characterized by the extensive horizontal transfer of genes, while *Yersinia pestis*, due to its well-characterized repertoire of genomic repeats, provides a good test case for MetaCarvel’s ability to detect repeats. Two synthetic datasets are used to evaluate the performance of MetaCarvel on more complex communities where the sequence of all the organisms in the mixture are known—the MBARC-26 dataset representing real sequencing data of a synthetic mixture of cells [27] and the simulated dataset created by the CAMI project [28]. Finally, we present the results obtained by analyzing real metagenomics datasets from the Human Microbiome Project [2].

### Effect of microbial mixtures on scaffolding

We compared the performance of MetaCarvel to that of OPERA-LG [24], using both single genomes and an increasingly complex mixture of genomes. We used reads from five different strains of *Acinetobacter baumanii* (NCBI Short Read Archive accessions SRR1008889, SRR1030406, SRR1019232, SRR1030403, and SRR1030473) and assembled them using both MEGAHIT [8] and metaSPAdes [7]. We chose *Acinetobacter baumanii* due to the availability of a high-quality reference and high-quality assemblies of multiple strains in public databases. These specific strains were selected because their assemblies were of high and similar quality and because they diverged sufficiently from each other to reveal the impact of strain variants on the quality of assembly and scaffolding.

**Fig. 1:**
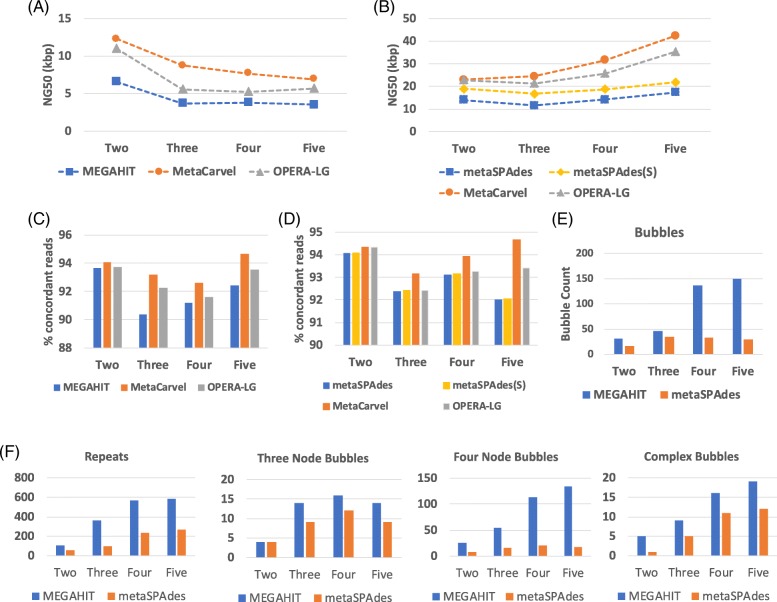
Scaffold statistics for *Acinetobacter baumannii* strain mixtures. **a** NG50 statistics when MEGAHIT contig assembly was used as an input for scaffolding methods. **b** NG50 statistics when metaSPAdes contig assembly was used as in input for scaffolding methods. metaSPAdes(S) denotes the scaffolds generated by inbuilt scaffolding module of metaSPAdes. The percentage of reads aligned concordantly when **c** MEGAHIT assembly was used as an input and when **d** metaSPAdes assembly was used as an input. **e** Number of bubbles detected by MetaCarvel for different input assemblies. **f** The count of different types of variants in *Acinetobacter* strain mixtures. Complex bubbles denote all the bubbles containing five or more nodes

To simulate the impact on scaffolding performance of increasing levels of genome heterogeneity among closely related organisms, we created increasingly complex mixtures comprising from one to five genomes. We aligned the paired reads to the resulting assemblies and used MetaCarvel and OPERA-LG to perform scaffolding. As expected, as more genomes are added to the mixture, the quality of the assembly degrades and so does the quality of the resulting scaffolds (Fig. 1a, b). Even in the case of the assembly of a single genome, scaffolding with MetaCarvel improves contiguity, albeit by only a small amount (13.31 kbp contig NG50 vs.18.51 kbp scaffold NG50 using MEGAHIT and 16.96 kbp contig NG50 vs. 18.99 kbp scaffold NG50 using metaSPAdes). The contiguity of the scaffolds generated by MetaCarvel substantially improves over the original assembly for the more complex samples. When compared to metaSPAdes scaffolds (generated using the scaffolding module built within this assembler), MetaCarvel’s scaffold contiguity was at least as good as metaSPAdes scaffolds for all mixtures (Fig. 1b). The contiguity of the scaffolds degrades slower than that of the scaffolds generated by OPERA-LG even as the contiguity of the underlying contigs created by MEGAHIT and metaSPAdes degrades rapidly with the increase in complexity of the mixture.

To measure the correctness of the assemblies, we computed the number of mate pairs mapped concordantly, that is, the mate pairs whose two ends are properly oriented with respect to each other and the distance between the paired reads is within the insert size limit implied by the library. This measure is correlated with assembly quality as misassemblies, or fragmented contigs and scaffolds, result in unmapped reads and discordant mate pairs. For all the mixtures and both assemblers, MetaCarvel scaffolds had the highest number of concordant mate pairs (Fig. 1c, d).

**Fig. 2:**
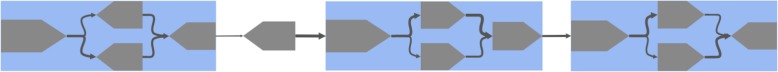
Variants detected in one of the components of *Acinetobacter baumanii* scaffold graph. In this component, we find all the non-terminal nodes in a bubble are more than 97% identical to each other and originate from two different strains of *Acinetobacter baumannii* genome

As the number of genomes in a mixture increased so did the number of genomic variants detected by MetaCarvel (Fig. 1e). The number of variants detected by MetaCarvel increased when adding more genomes to the mixture (Fig. 1f) across all the categories of features identified by the software: repeats, three-node bubbles (insertions/deletions), four-node bubbles (strain variations), and complex rearrangements (five or more node bubbles). A sample pattern of variation is shown in Fig. 2. In this example, the parallel contigs differed by about 3% nucleotide identity, a value larger than the amount of error tolerated by the assemblers. We observed that the number of variants detected by MetaCarvel was much higher when using MEGAHIT assemblies compared to metaSPAdes. However, the contiguity of scaffolds generated with metaSPAdes was higher than that of scaffolds relying on MEGAHIT.

### Detection of regions with high sequence variation

**Fig. 3:**
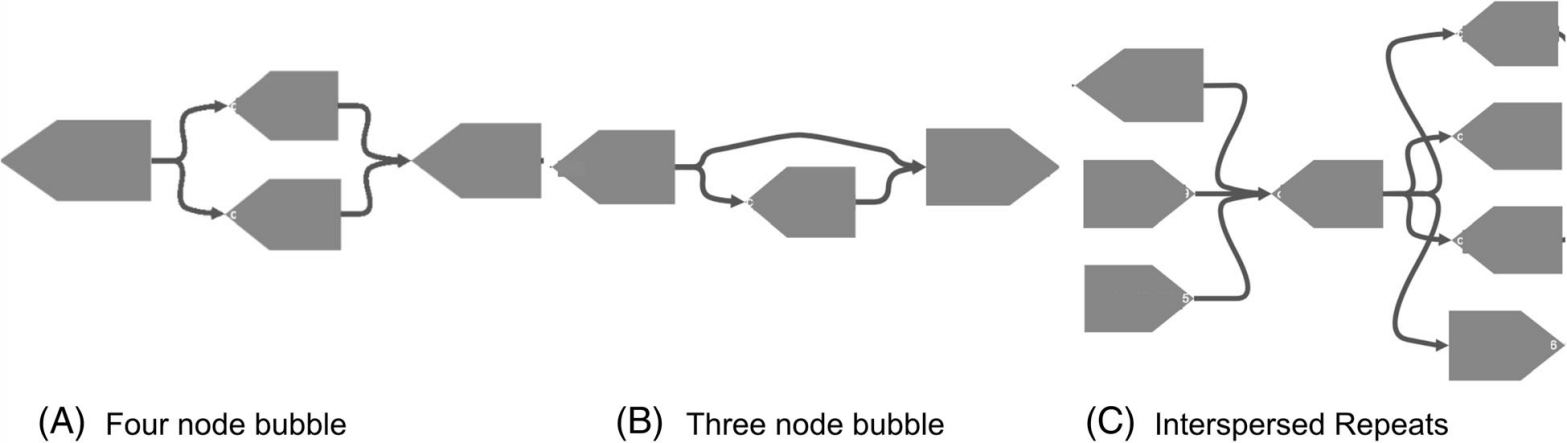
Different types of motifs detected by MetaCarvel. **a** Four-node bubbles denote the variation between very similar sequences. They can result in the graph due to the species with very high sequence similarity. **b** Three-node bubbles potentially represent gene gain/loss events and horizontal gene transfers. They are formed due to the insertion or deletion of chunks between two otherwise similar genomes. **c** Interspersed repeats in the graph are denoted by the nodes with high centrality and usually tangle the graph

To evaluate the accuracy of sequence variants (four-node bubbles, Fig. 3a) detected by MetaCarvel, we used reads from two strains of *Acinetobacter baumannii* genome that are distantly related (SRR1171982 and SRR1200567) [26]. We co-assembled the reads with MEGAHIT and ran MetaCarvel’s variant detection on the resulting assembly. We aligned the contigs to the *Acinetobacter baumannii* 1656-2 reference genome sequence (NCBI ID: NC_017162). The contigs which aligned at a same position in the reference genome were inferred to have originated from the true variants. MetaCarvel detected 191 variants in this graph, among which 184 overlapped with variants identified by alignment to the reference genome. In the remaining 7 variants which could not be validated using the strain 1656-2, the contigs from these variants were perfectly aligned to *Acinetobacter baumannii* strain AR_0078, *Acinetobacter baumannii* strain XH731, and *Acinetobacter baumannii* strain 15A34. For the remaining bubbles, the contigs in those bubbles did not align to any known strain of *Acinetobacter baumannii* with high identity, suggesting possible misassemblies. We also performed a similar analysis on a mixture of *Escherichia coli* K12 and *Escherichia coli* O83:H1 genomes. In this case, to flag a true variation, we check if contigs in a bubble are aligned to both the strains with high identity over at least 95% of their length. With this definition, 28 of 31 bubbles found by MetaCarvel matched actual variants, implying 90.3% precision.

### Accuracy of detecting insertions and deletions

To verify the accuracy of detecting insertion and deletions, we used MEGAHIT to co-assemble reads from two strains of *Escherichia coli* for which fully complete reference sequences are available: *Escherichia coli* K12 (NCBI sequence read archive accession: ERR022075) and *Escherichia coli* O83:H1 (NCBI sequence read archive accession: SRR6512538). We scaffolded the resulting assembly using MetaCarvel and flagged as predicted insertion/deletion events the three-node bubbles (see Fig. 3b) found within the resulting scaffolds. To characterize the true insertion and deletion events between these two *Escherichia coli* genomes, we aligned them to each other using NUCmer and extracted the regions flagged as “GAP” by the *dnadiff* utility from the MUMmer package [29]. We determined that a three-node bubble represented a true insertion/deletion event if the middle contig of the variant aligned within one of these regions. Of 126 three-node bubbles detected by MetaCarvel, 81 were found concordant with the insertion/deletion events identified by MUMmer (64.2% precision). A total of 194 contigs aligned to gap regions within the *E*. *coli* genomes, implying a specificity of 73.1%. Some of the false negatives (32) were due to the parameters used in MetaCarvel to eliminate low-quality edges in the graph, while the remaining false negatives were due to the fact that the insertion/deletion event coincided with other genomic phenomena, leading to a graph motif that was inconsistent with our definition of a three-node bubble.

### Effectiveness in detecting repeats

To determine the accuracy of interspersed repeat detection (Fig. 3c), we used reads from *Yersinia pestis* CO92 genome (Genebank ID: AL590842.1) as this genome has well characterized interspersed repeats [30]. We assembled the reads (SRA ID: SRR069183) using MEGAHIT and then scaffolded the assembly with MetaCarvel. To define a ground truth, we aligned the contigs to the *Yersinia pestis* genome using NUCmer [29] (with --maxmatch option) and flagged as repeats all contigs aligned at more than one location with at least 95% identity and 95% alignment length. The precision and recall of MetaCarvel’s repeat detection algorithm were 14.86% and 71.73% respectively. We compared this result to the algorithm used in OPERA-LG which detects repeats using sequence coverage alone (contigs with 1.5 times the average coverage of the genome are flagged as repeats). Within the same assembly of *Yersinia pestis*, OPERA-LG’s repeat finding approach has precision and recall of 9.06% and 67.39%, respectively (Table 1).

**Table 1:** Comparison of the accuracy of repeat detection in MetaCarvel and OPERA-LG on different datasets

Dataset	Method	True repeats	Predicted repeats	True positives	False positives	True negatives	False negatives	Precision (%)	Recall (%)
*Yersinia pestis*	OPERA-LG	46	353	31	322	904	15	9.06	67.39
	MetaCarvel	46	222	33	189	1037	13	14.86	71.73
MBARC-26	OPERA-LG	532	771	356	415	21,215	176	47.34	66.91
	MetaCarvel	532	454	438	16	21,614	94	96.47	85.33
CAMI (M)	OPERA-LG	3809	4568	2989	1579	154,936	820	65.43	78.74
	MetaCarvel	3809	4301	3317	984	155,531	492	77.12	87.08
CAMI (H)	OPERA-LG	12,666	27,625	7078	20,547	1,157,503	5558	25.62	56.01
	MetaCarvel	12,666	9219	7266	1953	1,176,067	5400	78.81	57.36

Further, we assessed MetaCarvel’s repeat detection accuracy on a synthetic metagenomic dataset (MBARC-26) described in Singer et al. [27]. This dataset (MBARC-26) consists of a mixture of 23 bacterial and three archaeal stains, across 10 different phyla and 14 classes, as well as a wide range of GC and repeat content. We assembled the reads using MEGAHIT, and the resulting contigs were aligned to the reference genomes using NUCmer (with --maxmatch option). In this case, the precision and recall of MetaCarvel’s repeat detection were 96.47% and 85.33%, respectively, compared to 47.34% and 66.91% for OPERA-LG (Table 1). The repeats missed by MetaCarvel had inconsistent read alignments and hence were not part of the scaffold graph. Of the 16 false positives obtained from MetaCarvel, 8 of them were marked with “high coverage node” as one of the features and 3 of them were marked based on high betweenness centrality (see the “Methods” section for details).

### Evaluation of scaffold quality using synthetic datasets

We evaluated MetaCarvel’s scaffold quality on the MBARC-26 dataset [27]. Due to the high depth of sequencing coverage and relatively low complexity of the mixture, the assembly of the full dataset resulted in large contigs and few opportunities for scaffolding algorithms to improve contiguity. Only 0.051% of mate pairs spanned the gap between contigs, thereby not providing linking information for scaffolding. To provide a more challenging situation, we downsampled the total number of reads 1000-fold. We assembled the downsampled data using MEGAHIT with default parameters. To derive linkages between contigs based on mate pair information, we aligned the reads to the assembled contigs using bowtie2 (with parameters -end-to-end -p 12) [31]. We then used MetaCarvel and OPERA-LG to scaffold these assemblies. Since we know the reference genome sequences for this dataset, we could use metaQUAST [32] to assess the accuracy of the resulting scaffolds. As seen in Table 1, MetaCarvel had fewer misassemblies and better contiguity than OPERA-LG, even in this relatively simple community.

We also assembled the data using metaSPAdes (with default parameters), an assembler specifically developed for metagenomic data that also includes a scaffolding module. We scaffolded metaSPAdes contigs with MetaCarvel and OPERA-LG and used metaQUAST to evaluate scaffold accuracy. As seen in Table 2, the number of misassemblies in MetaCarvel scaffolds was lower than that in OPERA-LG but higher than that in metaSPAdes scaffolds. MetaSPAdes scaffolds had fewer misassemblies because their scaffolding module is tightly coupled with the assembly module, hence uses more information obtained from the assembly graph to generate scaffolds. The contiguity of MetaCarvel scaffolds was better than that of both metaSPAdes and OPERA-LG scaffolds.

**Table 2:** Comparison of MetaCarvel with OPERA-LG on a synthetic metagenomics datasets

Dataset	Method	Number of scaffolds	Assembly size (Mbp)	Misassemblies	Largest scaffold (kbp)	Scaffolds > 50 kbp	Length at 1 Mbp (kbp)	Length at 10 Mbp (kbp)	Length at 50 Mbp (kbp)
MBARC-26	MEGAHIT + OPERA-LG	22,597	87.5	207	473.2	180	319.2	97.0	2.3
	MEGAHIT + MetaCarvel	19,879	88.0	99	287.3	177	287.3	154.3	9.1
	metaSPAdes + OPERA-LG	4931	98.7	148	2331.2	412	2055.2	944.0	177.1
	metaSPAdes + MetaCarvel	5137	99.8	68	199.1	298	1810.2	1125.0	199.4
	metaSPAdes scaffolds	5333	98.3	55	199.1	410	1810.2	925.6	158.3
CAMI medium	MEGAHIT + OPERA-LG	157,389	271.7	1669	854.1	527	825.4	404.6	73.1
	MEGAHIT + MetaCarvel	158,340	271.5	1793	854.1	520	825.4	418.2	74.5
CAMI high	MEGAHIT + OPERA-LG	1,188,757	1121.8	23,224	613.6	824	581.5	299.8	96.6
	MEGAHIT + MetaCarvel	1,185,065	1121.2	22,401	1089.6	847	613.6	304.5	101.8

### Evaluation using CAMI-simulated metagenome datasets

To further test the accuracy of MetaCarvel on complex simulated communities, we used the data for medium and high complexity metagenome communities released in CAMI challenge [28]. We assembled the reads in these datasets using MEGAHIT and used MetaCarvel and OPERA-LG for scaffolding. We were not able to run metaSPAdes on either of these datasets as the memory requirement exceeded 150 Gb. We used the reference genomes provided by the CAMI consortium to evaluate scaffold accuracy. On both medium and high complexity datasets, we observed that MetaCarvel’s repeat classification accuracy was better than OPERA-LG, although the recall was low for detecting repeats in the high complexity dataset (Table 1). In the medium complexity dataset, the contiguity was similar for OPERA-LG and MetaCarvel with the number of misassemblies lower for OPERA-LG (Table 2). In the high complexity dataset, MetaCarvel scaffolds were more contiguous with fewer misassemblies than OPERA-LG. This evaluation shows that MetaCarvel’s repeat detection and scaffolding works better on complex metagenomic communities than OPERA-LG.

### Evaluation using real metagenomics data

**Fig. 4:**
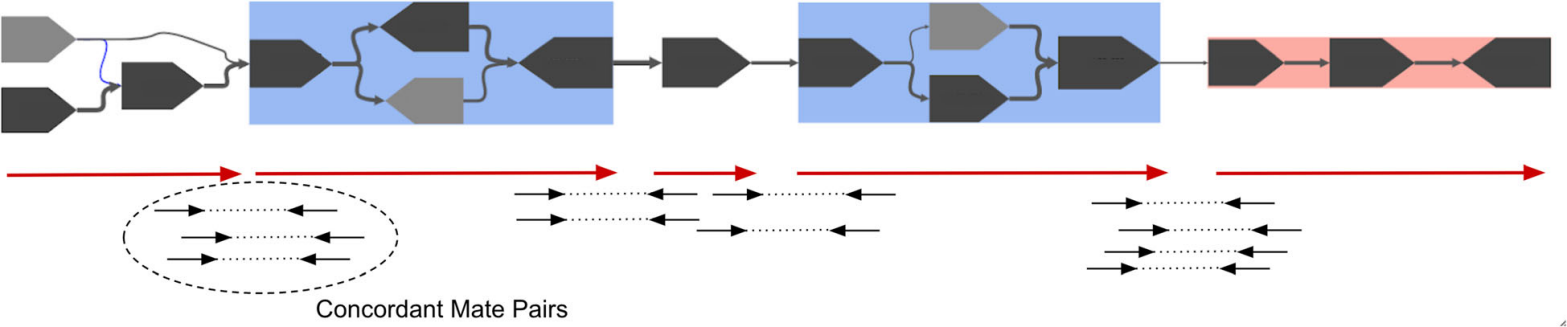
A component in the scaffold graph for the HMP stool sample. The highlighted nodes in the graph denote the path taken by MetaCarvel to generate the scaffold in this component, and the red lines denote the scaffolds generated by OPERA-LG. It can be observed that at the boundaries of the regions of variation, OPERA-LG breaks scaffolds thereby not using the information provided by the paired-end reads to generate contiguous and accurate scaffolds

We tested the effectiveness of MetaCarvel on four samples from the Human Microbiome Project (HMP) [2]. We chose two stool samples (SRS020233, SRS049959), one supragingival plaque sample (SRR2241598), and a posterior fornix sample (SRS024310). The stool samples represent complex communities and have high depths of sequencing coverage and the plaque sample has lower complexity but relatively high coverage, while the posterior fornix has a lower depth of coverage due to the high level of host contamination (more than 80% human DNA) [2]. Table 3 shows the comparison of different scaffolding approaches on these samples. Since the composition of these samples is unknown, we could not use reference-based methods to evaluate scaffold accuracy. Instead we computed the number of mate pairs that map concordantly to the resulting scaffold. For all the samples, MetaCarvel had a higher number of concordant mate pairs compared to OPERA-LG when the MEGAHIT assembly was used. Even when scaffolding metaSPAdes assemblies, MetaCarvel had the highest number of concordant mate pairs. Also, the total number of concordant mate pairs was higher for both OPERA-LG and MetaCarvel scaffolds when using the MEGAHIT assembly compared to the metaSPAdes assembly as an input. Since a metagenomic assembly does not have a known total genome size, the use of measures such as N50 and NG50 (commonly used for comparing the contiguity of isolate genome assemblies) is not appropriate. To assess the contiguity of scaffolds in a way that can be compared across assemblies of a dataset, we first sort the scaffolds in decreasing order of their lengths. Then, we start adding the lengths of scaffolds until a particular target length is reached (1 Mbp, 10 Mbp, and 50 Mbp in our case). The length of the scaffold at which the total sum of the length-sorted scaffolds exceeded the target length becomes the statistic to assess contiguity of the scaffolds. In other words, “size at 10 Mbp” represents the longest length *L* such that the sum of all scaffold lengths longer than *L* adds up to 10 Mbp or above. In most cases, MetaCarvel scaffolds had the highest contiguity. Particularly, the best results were obtained by scaffolding with MetaCarvel the contigs that were generated by metaSPAdes. The high contiguity and the high number of concordant mate pairs in MetaCarvel scaffolds can be attributed to its ability to resolve the bubbles in the connected components and generate the scaffolds which pass through the bubbles, whereas OPERA-LG broke the scaffolds where there was a boundary between a variant and a linear path (Fig. 4). As a result, the mate pairs spanning these junctions were not explained by OPERA-LG scaffolds.

**Table 3:** Comparison of reference-free assembly statistics for real metagenomic datasets generated in the HMP project

Dataset	Method	#Scaffolds	Assembly size (Mbp)	#Scaffolds > 50 kbp	Largest scaffold (kbp)	#Concordant mate pairs	Length at 1 Mbp (kbp)	Length at 10 Mbp (kbp)	Length at 50 Mbp (kbp)
SRS049959	OPERA-LG	198,206	273.2	473	530.1	97,428,296 (82.1%)	258.6	126.3	38.9
	MetaCarvel	108,437	277.0	487	518.2	98,107,950 (85.5%)	356.7	154.1	39.5
	metaSPAdes	98,318	268.3	489	476.5	91,870,816 (80.0%)	422.8	164.1	44.8
	OPERA-LG (M)	97,486	267.7	518	476.5	91,948,044 (80.1%)	405.1	162.2	47.0
	MetaCarvel (M)	98,073	268.1	492	868.8	92,183,496 (80.3%)	749.8	211.0	49.2
SRS020233	OPERA-LG	128,250	279.8	393	381.6	91,464,778 (84.9%)	286.5	139.7	35.4
	MetaCarvel	141,438	282.5	421	430.2	92,077,670 (86.9%)	368.6	154.3	37.8
	metaSPAdes	122,613	279.6	437	573.8	91,577,014 (85.9%)	351.9	163.9	40.9
	OPERA-LG (M)	122,143	279.9	459	573.8	91,622,740 (85.3%)	372.1	158.3	42.4
	MetaCarvel (M)	122,776	280.8	471	587.2	91,840,800 (85.3%)	584.7	187.1	44.7
SRR2241511	OPERA-LG	631	284.4	5	962.9	12,618,990 (83.9%)	19.3	NA	NA
	MetaCarvel	533	285.9	6	126.1	12,665,752 (84.2%)	27.7	NA	NA
	metaSPAdes	774	334.5	4	570.3	13,838,686 (91.0%)	20.6	NA	NA
	OPERA-LG (M)	733	334.3	6	124.9	12,875,136 (85.6%)	20.9	NA	NA
	MetaCarvel (M)	652	335.4	11	126.3	12,910,216 (85.9%)	37.74	NA	NA
SRR2241598	OPERA-LG	60,601	117.6	75	217.4	19,423,228 (51.6%)	148.3	35.1	4.1
	MetaCarvel	56,503	119.1	100	319.2	20,047,708 (54.0%)	184.2	46.6	5.7
	metaSPAdes	48,403	113.6	102	417.4	16,771,928 (45.2%)	206.9	46.5	6.2
	OPERA-LG (M)	43,908	109.4	105	282.9	16,749,600 (45.1%)	206.9	47.9	6.6
	MetaCarvel (M)	42,927	110.2	190	417.4	16,893,882 (45.5%)	336.43	97.5	8.7

For the concordant mate pairs, the number in the parenthesis denotes the percentage of total read pairs mapped concordantly to scaffolds. In methods, (M) denotes scaffolds generated using metaSPAdes contigs as input to MetaCarvel and OPERA-LG

### Effect of sequencing coverage on metagenome scaffolding

To assess the impact of sequencing depth on the scaffolding algorithm, we downsampled the synthetic mock community dataset MBARC-26 [27] to a range of total number of reads between 2 million and 14 million and used the resulting assemblies as input for OPERA-LG, MetaCarvel, and metaSPAdes. Note that the input assembly for OPERA-LG and MetaCarvel was generated using MEGAHIT, while metaSPAdes scaffolds were generated using the built-in scaffolding module. As expected, at low depths of coverage, the contig and scaffold contiguity was poor but improved as more reads were added (Table 4).

**Table 4:** Performance comparison of different scaffolders based on varying the sequencing depth on the MBARC-26 dataset.

Input size (millions of reads)	Metric	No. of scaffolds	Length at 1 Mbp (bp)	Length at 10 Mbp (bp)	Length at 50 Mbp (bp)	CPU time	Peak memory (GB)
2	OPERA-LG	29,831	988,539	90,227	719	44 s	2.2
	metaSPAdes	61,592	594,287	92,217	783	NA	8.2
	MetaCarvel	29,883	699,981	90,014	718	58 s	2.1
4	OPERA-LG	22,952	1,257,853	168,019	4393	2 min 16 s	2.8
	metaSPAdes	49,199	1,635,634	190,132	3823	NA	10.1
	MetaCarvel	23,003	1,257,853	168,390	4374	2 min 48 s	3
8	OPERA-LG	21,866	1,257,855	393,755	34,351	3 min 13 s	4
	metaSPAdes	39,460	1,635,634	190,132	31,823	NA	14.3
	MetaCarvel	23,003	1,223,449	423,739	32,331	3 min 47 s	3.8
10	OPERA-LG	21,413	1,257,855	402,996	50,874	8 min 01 s	5
	metaSPAdes	35,754	1,635,634	478,925	52,165	NA	22.5
	MetaCarvel	21,033	1,332,109	418,821	49,839	10 min 41 s	5.2
14	OPERA-LG	18,370	1,461,964	676,339	72,581	14 min 08 s	8.1
	metaSPAdes	29,298	1,635,789	668,856	78,337	NA	28.1
	MetaCarvel	18,281	1,463,318	686,311	73,522	13 min 14 s	7.4

The runtime for metaSPAdes is not mentioned (marked NA) since we cannot separate the assembly from the scaffolding steps. Maximum contig size is the same for OPERA-LG and MetaCarvel because the same input assembly was used as input to them

### Computational requirements of MetaCarvel

The computational requirements of MetaCarvel mainly depend on the size of the assembly graph, specifically the number of contigs in the assembly and the number of links between these contigs. The input assembly for the MBARC-26 dataset (~ 20 million reads) had 19,326 contigs, and its scaffolding required peak memory of 8.2 GB with the CPU runtime of 18 min. For the scaffolding of stool sample (SRS049959, ~ 54 million reads), the number of contigs in the input assembly was 214,985 and its scaffolding required peak memory of 38.7 GB and CPU runtime of 88 min. Table 4 lists the runtime and memory requirements for scaffolding with different number of reads. The runtime and memory requirements increase as a greater number of reads are used. The growth is supra-linear because the runtime of scaffolding algorithm mainly depends on the number of edges in the scaffold graph, which can grow quadratically in the worst case. The runtime and memory requirements for OPERA-LG and MetaCarvel were comparable for all the sequencing coverages.

## Discussion

We described a stand-alone metagenomics variant detection and scaffolding method MetaCarvel and showed its effectiveness on synthetic and real metagenomics datasets of varying complexity. Unlike most of the existing scaffolders which only output linearized sequences of scaffolds, MetaCarvel outputs a list of variants along with the graph used to call variants. This information can help biologists to explore interesting graph patterns within the assembly and investigate the biological implications of the corresponding genomic variants.

To allow a quantitative evaluation of variant detection, we focused our validation on simple types of genomic variants that involve three or four contigs. MetaCarvel does detect more complex variants, which are, however, difficult to validate in an automated fashion. This functionality sets MetaCarvel apart from other tools available for identifying strain variants in microbial communities, tools which primarily rely on reference genomes or conserved genes to characterize microbial strains. The approach taken by MetaCarvel is complementary to approaches based on marker genes, such as StrainPhlAn [18]. The combination of the two approaches represents a promising direction for future research, leading to effective approaches for characterizing novel genomic fragments while placing them within the context of the fine grained taxonomic information derived from marker genes.

The effectiveness of the approach implemented in MetaCarvel critically depends on the data available to the scaffolding module. Note that the lack of contiguity manifests due to two reasons: (i) lack of contiguity in the assembly used as an input to the scaffolding algorithm and (ii) lack of linking information available for scaffolding algorithms to join contigs into scaffolds. MetaCarvel can only detect variants if the corresponding contigs are covered at high enough depth and if mate pairs or other information provide links between adjacent contigs. The analysis is also greatly improved if the underlying assembly is conservative—assemblers that aggressively attempt to “smooth out” genomic variants in order to obtain long genomic contigs end up removing exactly the information that MetaCarvel is designed to detect. We, thus, suggest that scientists interested in strain variation explore multiple assemblies of datasets, using different metagenomic assemblers run with different parameter choices, rather than relying on published assemblies or using the most popular assembler run with default parameters.

>Beyond the choice of parameters for the assembler used to generate the input to MetaCarvel, users can also control the number of links required to construct an edge between adjacent contigs. If this threshold is low, the graph can have many spurious edges, leading to longer runtime, reducing the accuracy of repeat detection, and complicating variant discovery. If this threshold is high, the graph becomes disconnected leading to a degraded ability to discover variants, and low scaffold contiguity. Although the repeat detection procedure used in MetaCarvel does not expose any parameters to the end user, its accuracy depends on the number of features that provide evidence of contig’s repetitiveness—features that are also related to the density of links in the scaffold graphs.

In closing, we would like to stress that the study of strain variation within microbial communities is in its infancy, in no small part due to the relative dearth of appropriate datasets and analytic tools. Tools such as MetaCarvel, StrainPhlAn, and others are just a first step towards the development of an effective toolkit for the discovery and characterization of genomic variants. Of particular interest will be the development of approaches able to infer the functional implications of strain variants, ultimately leading to a better understanding of the principles underlying microbial adaptation and community structure.

## Methods

**Fig. 5:**
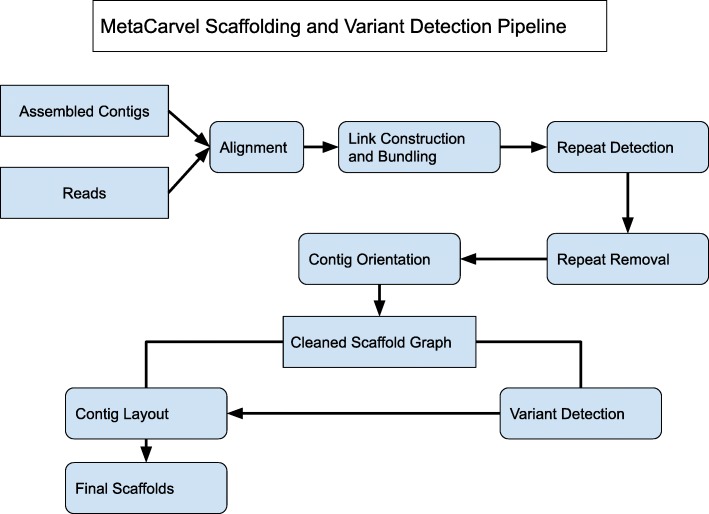
Overview of the MetaCarvel pipeline: First, reads are aligned to assembled contigs. Using these alignments, a scaffold graph is constructed by bundling the link implying same contig orientation. In this graph, repeats are identified and removed. In the repeat removed scaffold graph, first variants are detected, and variant-aware contig layout is performed to generate final scaffolds. The output of the MetaCarvel pipeline is the sequences for scaffolds and the list of variants found in the scaffold graph

MetaCarvel operates as a series of discrete steps that construct and progressively refine a graph linking together assembled contigs with the information provided by mate pair or paired-end reads (Fig. 5). Currently, we determine the links between contigs by remapping the paired reads to an assembly constructed by a metagenomic assembler. This step is necessary as current assemblers do not provide information about the placement of individual reads within the assembled contigs. When such information is available, MetaCarvel can directly use it, currently accepting the information in SAM/BAM formats.

### Contig graph construction

MetaCarvel begins by aligning paired-end reads to assembled contigs using a standard short read aligner such as BWA [33] or bowtie2 [31]. The reads are aligned in a single end mode to avoid biasing alignments based on the pre-specified library insert size. Rather, the library size is recomputed by MetaCarvel and errors in read pairing are identified during the scaffolding process. Using the alignments of reads to contigs, a contig graph is created where the nodes are contigs and edges between adjacent contigs indicated that one or more paired-end reads span the gap between the corresponding contigs. We first re-estimate the library size (mean and standard deviation) by considering pairs where both reads in the pair are aligned to the same contig. To account for divergent estimates of the distance between adjacent contigs, we compute the maximal set of links that are consistent with each other and that imply a similar distance. Finding such a set of consistent links is equivalent to finding a maximal clique in an interval graph as described in [34]. Once the set of mutually consistent links is identified, they are “bundled” into a single representative link. The mean and standard deviation for this link is computed using a method described in Huson et al. [35]. The weight of this link is given by the number of read pairs which were bundled while constructing the link. Bundling of links gives a single value for mean and standard deviation for the implied distance between a pair of contigs.

### Repeat identification

To avoid the ambiguities caused by genomic repeats during scaffolding, we first identify repetitive contigs and remove them from the contig graph along with all the edges incident on them. We use several properties of the graph and contigs to determine the contigs that could confound the scaffolding process [36]. First, we calculate the sequencing coverage and degree for all the contigs in the graph. Then, we assign a unique orientation to each contig in the graph using an algorithm described in more detail in the next section. This algorithm removes edges from the graph that prevents the assignment of a consistent orientation to contigs. For example, if a contig is assigned the forward orientation, then all the links implying the reverse orientation are removed. For each contig, we count the number of invalidated edges. We also flag links in the contig graph that connect contigs with significantly different depths of coverage. We track how many such “skewed” links are incident on each contig. A more detailed description of how these features are computed can be found here [36].

**Fig. 6:**
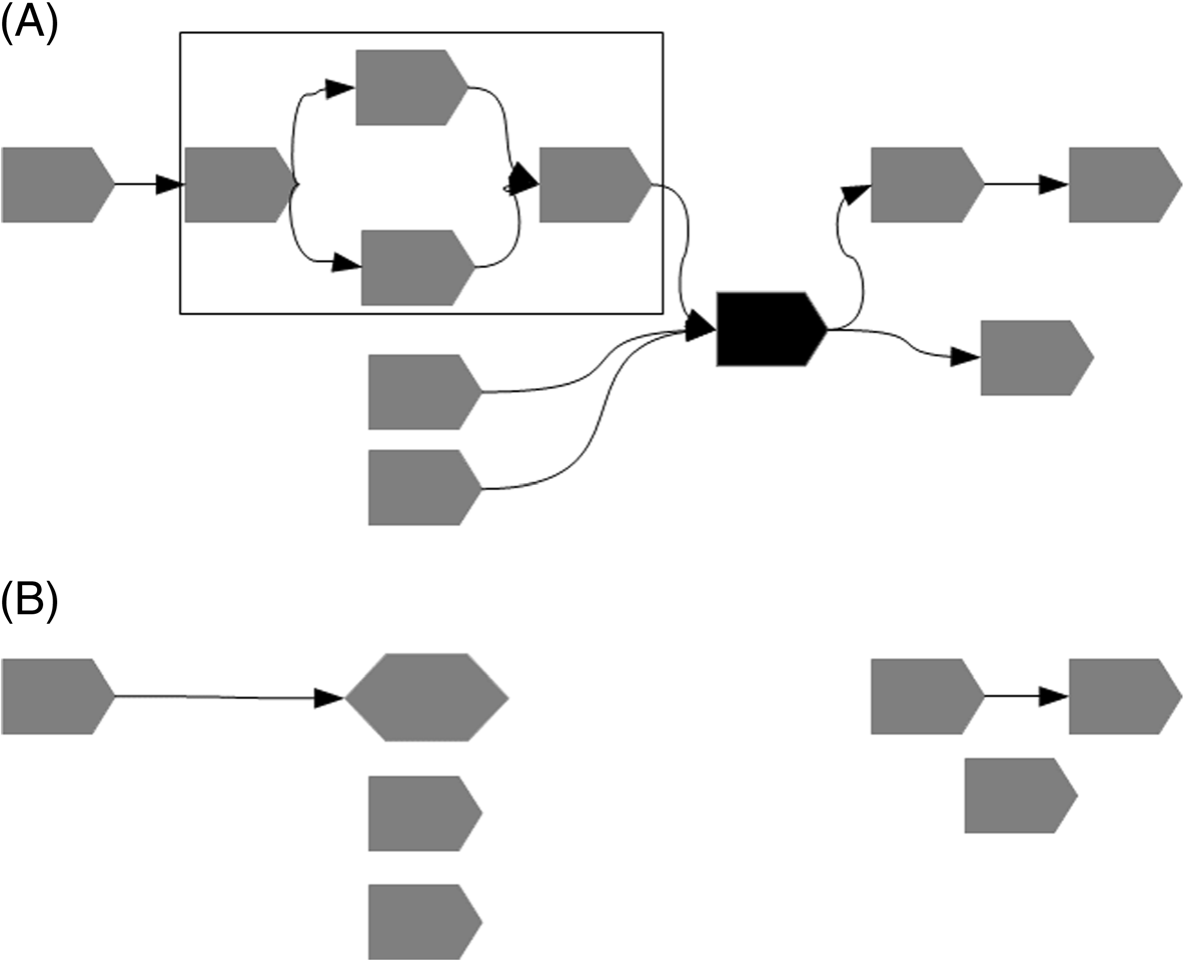
The impact of repeat detection and variant discovery on the scaffolding graph. **a** A scaffolding graph containing a four-node bubble (highlighted by a box) and a repeat (node shaded black). **b** After the removal of the repeat, the graph becomes disconnected, simplifying the discovery of variants (the collapsed four-node bubble is shown as an elongated hexagon) and simple chains of contigs (the remaining two edges in the graph)

For each of the features described above (depth of coverage, node degree, incident edges invalidated during the orientation phase, skewed edges), we flag the contigs that occur within the upper quartile among all contigs. Any contig that is flagged according to at least three of the criteria listed above is marked as a repeat and removed. After removing these contigs, we also remove contigs with a high betweenness centrality measure (the number of shortest paths passing through a node in a graph) [37]—specifically the contigs that have a betweenness centrality higher by more than 3 standard deviations from the mean betweenness centrality for the assembly graph. Since the computation of betweenness centrality is computationally expensive (*O*(*N*^3^) for *N* contigs), we use an approximation algorithm [38] which runs in linear time, thereby scaling to large graphs obtained from the complex metagenomic samples. The impact of the node removal on the structure of the scaffolding graph is shown in Fig. 6.

### Orientation

The contig graph is bidirected because each contig in the graph can originate from either forward or reverse DNA strand. To make this graph directed, we need to assign a unique orientation to each contig. The edges in the graph are of two types: “same” when adjacent contigs have the same orientation and “different”, otherwise. If the graph has a cycle that contains an odd number of “different” edges, then it is impossible to assign a consistent orientation to contigs in that cycle without discarding at least one edge from the cycle. Our objective is to minimize the number of edges to be removed from the graph in order to allow a consistent orientation for all contigs. Finding such a minimum set is equivalent to finding a maximal bipartite subgraph—an NP-Hard problem [39]. We use the greedy algorithm described in Kelecioglu et al. [40] that achieves a two-factor approximation and runs in *O*(*V + E*) time (*V*—the number of contigs, *E*—the number of edges connecting these contigs). Briefly, we assign an arbitrary orientation (forward or reverse) to a starting contig, then proceed to orient all contigs adjacent to it. While assigning an orientation to a contig, we pick an orientation in such a way that it agrees with the majority of its already oriented neighbors (in terms of edge weights supporting that orientation). Once we assign an orientation to a contig, we invalidate any links that disagree with the chosen orientation. We continue in a breadth-first manner and assign an orientation to all the contigs.

### Graph simplification and variant detection

A typical metagenomic sample contains closely related genomes or closely related strains of the same organism which result in a complex bubble-like pattern in the graph. Identifying complex variants in the graph takes exponential time in the number of nodes, thereby making variant identification extremely slow on large and complex metagenomics samples. To identify variants in the graph efficiently, we first decompose the oriented contig graph into its biconnected components using the Hopcroft-Tarjan algorithm [41]. This algorithm takes *O*(*V+ E*) time. We further decompose each biconnected component into triconnected components by computing SPQR tree data structures [42, 43]. The SPQR tree for a graph denotes a hierarchical decomposition of biconnected components of a graph into its triconnected components. We use the implementation of SPQR trees provided in the Open Graph Drawing Framework (OGDF) [44] which runs in linear time *O*(*V + E*). Since the SPQR tree data structure is only defined for undirected graphs, we need to check whether the components identified within the tree are consistent with the orientation of the edges of the assembly graph. We rely on the algorithm used in Marygold [20]: for each graph component identified between a pair of separation nodes within the SPQR tree, we check that all paths starting at the source node can reach the sink node of the component using a directed path. Components that fail this check are eliminated from further consideration. Once valid source-sink pairs and variants are identified, each component (complex graph “bubble”) is collapsed into a supernode. The incoming and outgoing edges from the source and sink respectively for the variants are assigned to its supernode. This simplifies the graph structure by a large extent thereby masking the complexities caused by the variants in the sample.

The graph components we identify are also reported by MetaCarvel as putative strain variants, allowing further analysis. From among the patterns identified, we have focused the analysis in this paper on three simple patterns (refer to Fig. 3).

#### Three-node bubbles

Three-node bubbles in the graph correspond to putative gene gain/loss events in the genome, hence, are important from the biological point of view. These bubbles can be easily found from the validated bubbles of size 3.

#### Four-node bubbles

Four-node bubbles correspond to putative variation between the genomes of related strains within a sample. Like three-node bubbles, they can also be easily characterized within the validated bubbles obtained during the bubble collapsing step.

#### Interspersed repeats

Interspersed repeats are natively detected and flagged by the repeat detection procedure described above.

### Generation of linear scaffolds

Once we simplify the graph by collapsing bubbles into supernodes, we generate the scaffold sequences through a linear traversal of the graph. We first create an auxiliary graph G’(V’,E’) from the original graph G(V,E), as follows. We create two nodes for each contig, one for the 5′ end and one for the 3′ end, connected by an edge that matches the orientation of the corresponding contig. The edge weights for E’ is the bundle sizes (number of mate pairs supporting that edge). The edges between the 5′ and 3′ ends of same contigs are not added at this stage. We then compute a weighted maximal matching [45] in G’. After we compute a weighted maximal matching, we remove nodes and edges present in that matching and repeat the matching process on the remaining nodes and edges until all nodes in G’ are matched. In each maximal matching, we add edges between the 5′ and 3′ ends of each contig present in that matching. This defines a unique linear path in G’ and spells out a scaffold. We note that supernodes (collapsed regions of strain variation) can be part of the linear path constructed from the scaffold graph. Since each variant is a directed acyclic graph (DAG), we compute the highest weighted path from source to sink within each supernode using a dynamic programming algorithm. This path is then merged within the global linear path to define the linearized scaffold. For each supernode, we also output additional source to sink paths as alternate variants by iteratively removing edges that were previously reported.

## Additional file

Additional file 1: Review history. (DOCX 22 kb)

## Data Availability

All the data used for validation is publicly available and downloaded from NCBI databases [2, 26–28]. The source code describing our methods is freely available under the MIT license at Github [46] and Zenodo [47].
