# Supplementary material for: MetaCarvel: linking assembly graph motifs to biological variants
Source: Genome Biol. 2019 Aug 26;20:174. doi: 10.1186/s13059-019-1791-3 (PMC6710874; doi:10.1186/s13059-019-1791-3)
Supplement: Supplementary file 1 — Review history. (DOCX 22 kb) [file 13059_2019_1791_MOESM1_ESM.docx]

Review History

**First round of review**

**Reviewer 1**

**Were you able to assess all statistics in the manuscript, including the appropriateness of statistical tests used? No**

**Were you able to directly test the methods? No**

**Comments to author:**

The manuscript describes MetaCarvel, a scaffolder reconstructing indels, SNPs and repeat-like variations from pangenome-like consensus assemblies of multiple strains generated from metagenomic data by current mg assemblers. This is a very interesting and timely innovation required by the field. The method is evaluated on several multi-strain data sets and a sequences from a mock community. In terms of scaffold quality, MetaCarvel was compared to the OPERA-LG as well as the metaSPAdes scaffolding methods, reporting a mid-range performance. The code is freely available, and comes with a good quality readme, installation and usage instructions.

Comments:

- the description of the methodology could be substantially improved by adding several illustrations on the utilized steps. At present, it is difficult to grasp in detail, unfortunately.

Performances in detecting SNPs, indels and repeats should also be evaluated on more complex metagenome benchmark data sets with strain-level divergence, e.g. from CAMI, not only on two-strain pangenome mixtures, to have an impression of its performance on more realistic data than what seems to have been used by the authors so far.

-The description of the evaluation on different strain mixutres has redundancies, and could be shortened and moved to methods.

- It would be nice to assess performance in detecting snps, indels and repeats on all three of these data sets, in addition to the evaluation suggested before, for a more comprehensive performance assessment. Furthermore, can the authors clarify on what basis they selected genomes for their evaluation - highest quality of the underlying assemblies would be ideal.

-since the manuscript is submitted to a journal with a broad readership, it would be nice to demonstrate some more biological insights. For instance, by visualizing into which gene regions SNPs fall or adding another application where pangenome data of a pathogen evolving under positive selection is analysed - SNPs should be detected in genes under known directional selection (of course, they should also be verified using the approach the authors take for the other multi-strain data sets they analyse.

-Specificity values given in table 1 (i.e. precision) seem very misleading - what is reported is not specificity, usually = TP/(TP +FP), but something else. Specificity should be 33/(33+189), not 84.6%. Could the authors resort to calculating precision/recall/F1-score, for instance, throughout? This can be done for both classes (i.e. repeats/non-repeats etc), if necessary.

Minor Comments:

-page numbers would be helpful!

-add definitions and unify nomenclature for the performance metrics. There is precision, recall, and specificity used at various places. Specificity, e.g. in table 1, is misleading, as this is not how it is normally reported - suggests mcuh better specificity than the method has in repeat detection.

-the text references Figure 6 but there is no Figure 6, it probably should be Figure 4.

-figures 3-5 have small text written on the contigs which is impossible to read and the highlighting in Figure 5 could be more obvious.

-other parts of the evaluation are not easy to understand. The assessment of the continuity states: "To assess the contiguity scaffolds, we sorted the scaffolds in decreasing order of their lengths and summed the length of the scaffolds until a particular length was reached (1 Mbp, 10 Mbp, and 50 Mbp in our case)". This needs a more detailed description, it sounds like the length of the scaffolds summing up to 1 Mbp should always be 1Mbp, but this is certainly not what this metric tries to measure (and Tables 2 and 3 describe something different).

-a link to the GitHub repository should be made available.

-the MBARC data set (Singer et. al.[32]) is introduced only the second time it is introduced.

**Reviewer 2**

**Were you able to assess all statistics in the manuscript, including the appropriateness of statistical tests used? Yes: All statistics seem to be reported in the manuscript.**

**Were you able to directly test the methods? Yes**

**Comments to author:**

The paper by Jay Ghurye et al. presents MetaCarvel, a novel variant-aware metagenomic scaffolder. The tool combines new strategies for repeat detection with graph analytics for variant discovery. The software is validated experimentally on simulated and real meta genomes and compared with an existing method (OPERA-LG). The tool is freely available in GitHub (<https://github.com/marbl/MetaCarvel>).

The topic involved in the paper is suitable for publication in this journal.

The paper is well written and well structured.

The methodological development seems sounded and interesting for me.

However, in my opinion the experimental validation needs to be improved before a possible publication:

1. Several existing scaffolding tools are already available. Although they are discussed in the literature survey part of the paper, they are not used as comparison methods in the experimental validation section. Only one tool (OPERA-LG) is considered for this purpose. An extension of the comparison to other existing tools should be performed, including the Bambus 2 tool developed by the same authors.

2. The proposed tool should be also validated on benchmark datasets. As example, I suggest considering the CAMI dataset (https://www.nature.com/articles/nmeth.4458)

3. It is not clear to me if the developed strategy depends from some free parameters (but it seems so looking at the webpage https://github.com/marbl/MetaCarvel). Please discuss in the text how they may influence the results.

4. A key point in the performance of the proposed tool is represented by the sequencing depth. I think you should evaluate this aspect in the manuscript in a more systematic way.

5. What in terms of computational load of the proposed tools? Please report some empirical evaluations.

**Authors Response**

**Point-by-point responses to the reviewers’ comments:**

Reviewer #1: The manuscript describes MetaCarvel, a scaffolder reconstructing indels, SNPs and repeat-like variations from pangenome-like consensus assemblies of multiple strains generated from metagenomic data by current mg assemblers. This is a very interesting and timely innovation required by the field. The method is evaluated on several multi-strain data sets and a sequences from a mock community. In terms of scaffold quality, MetaCarvel was compared to the OPERA-LG as well as the metaSPAdes scaffolding methods, reporting a mid-range performance. The code is freely available, and comes with a good quality readme, installation and usage instructions.

*We thank the reviewer for positive feedback and detailed review of the manuscript.*

Comments:

- the description of the methodology could be substantially improved by adding several illustrations on the utilized steps. At present, it is difficult to grasp in detail, unfortunately.

*We have updated the text describing the algorithm to make it easier to follow. We also have added figures highlighting the effect of repeat removal and variant collapsing on the scaffold graph. We have also provided additional references to publications that have previously described the various steps in the scaffolding process.*

Performances in detecting SNPs, indels and repeats should also be evaluated on more complex metagenome benchmark data sets with strain-level divergence, e.g. from CAMI, not only on two-strain pangenome mixtures, to have an impression of its performance on more realistic data than what seems to have been used by the authors so far.

*We have run MetaCarvel and OPERA-LG on CAMI medium and high complexity datasets and shown scaffolding performance. The results are updated in Tables 1 and 4.*

-The description of the evaluation on different strain mixtures has redundancies, and could be shortened and moved to methods. - It would be nice to assess performance in detecting snps, indels and repeats on all three of these data sets, in addition to the evaluation suggested before, for a more comprehensive performance assessment. Furthermore, can the authors clarify on what basis they selected genomes for their evaluation - highest quality of the underlying assemblies would be ideal. -since the manuscript is submitted to a journal with a broad readership, it would be nice to demonstrate some more biological insights. For instance, by visualizing into which gene regions SNPs fall or adding another application where pangenome data of a pathogen evolving under positive selection is analysed - SNPs should be detected in genes under known directional selection (of course, they should also be verified using the approach the authors take for the other multi-strain data sets they analyse.

*We have updated the text to clarify the evaluation, including by adding an overview to the results section.  We note that MetaCarvel does not identify single SNPs, rather our focus is on large structural variants or regions with high SNP density. We are not aware of an appropriate data set for performing the analysis you propose.  We are in the process of evaluating the biological implications of MetaCarvel's output, however including those results would substantially expand the scope of this manuscript. We have opted, instead, to focus on the technical validation of the approach in this manuscript.*

-Specificity values given in table 1 (i.e. precision) seem very misleading - what is reported is not specificity, usually = TP/(TP +FP), but something else. Specificity should be 33/(33+189), not 84.6%. Could the authors resort to calculating precision/recall/F1-score, for instance, throughout? This can be done for both classes (i.e. repeats/non-repeats etc), if necessary.

*We have updated table 1 to list precision and recall. We have also updated the text to reflect these changes.*

Minor Comments:

-page numbers would be helpful!

*We have added page numbers to the updated manuscript.*

-add definitions and unify nomenclature for the performance metrics. There is precision, recall, and specificity used at various places. Specificity, e.g. in table 1, is misleading, as this is not how it is normally reported - suggests mcuh better specificity than the method has in repeat detection.

*We have addressed this specific issue and updated table 1 and corresponding text to reflect the changes.*

-the text references Figure 6 but there is no Figure 6, it probably should be Figure 4.

*Thank you for pointing this out. We have correctly referred figures in text.*

-figures 3-5 have small text written on the contigs which is impossible to read and the highlighting in Figure 5 could be more obvious.

*We have changed the figures to remove node names (the text that was written on the contigs) as they are irrelevant to the information we intend to convey.*

-other parts of the evaluation are not easy to understand. The assessment of the continuity states: "To assess the contiguity scaffolds, we sorted the scaffolds in decreasing order of their lengths and summed the length of the scaffolds until a particular length was reached (1 Mbp, 10 Mbp, and 50 Mbp in our case)". This needs a more detailed description, it sounds like the length of the scaffolds summing up to 1 Mbp should always be 1Mbp, but this is certainly not what this metric tries to measure (and Tables 2 and 3 describe something different).

*We have updated the text that describes the contiguity statistic.*

-a link to the GitHub repository should be made available.

*The link to the repository and license details are provided at the end of the introduction.*

-the MBARC data set (Singer et. al.[32]) is introduced only the second time it is introduced.

*Thank you for catching this error introduced by reorganization of the text.  We have now described the data set the first time it is mentioned.*

Reviewer #2: The paper by Jay Ghurye et al. presents MetaCarvel, a novel variant-aware metagenomic scaffolder. The tool combines new strategies for repeat detection with graph analytics for variant discovery. The software is validated experimentally on simulated and real meta genomes and compared with an existing method (OPERA-LG). The tool is freely available in GitHub (<https://github.com/marbl/MetaCarvel>).

The topic involved in the paper is suitable for publication in this journal. The paper is well written and well structured. The methodological development seems sounded and interesting for me.

*We thank the reviewer for the positive feedback.*

However, in my opinion the experimental validation needs to be improved before a possible publication:

1. Several existing scaffolding tools are already available. Although they are discussed in the literature survey part of the paper, they are not used as comparison methods in the experimental validation section. Only one tool (OPERA-LG) is considered for this purpose. An extension of the comparison to other existing tools should be performed, including the Bambus 2 tool developed by the same authors.

*Most of the existing tools for scaffolding work with single genome. Amongst these tools,  OPERA-LG was shown to be the one giving better results based on Mandric et al. Hence we chose it for the comparison. We did not include Bambus2 in the comparison because it is no longer actively maintained by its developers (*[*https://genomeinformatics.github.io/projects/*](https://genomeinformatics.github.io/projects/)*) and uses obsolete file formats which are no longer used in the genome assembly research community. Without extensive code changes, Bambus 2 cannot be applied to modern data.*

2. The proposed tool should be also validated on benchmark datasets. As example, I suggest considering the CAMI dataset (<https://www.nature.com/articles/nmeth.4458>)

*We have run MetaCarvel and OPERA-LG on CAMI medium and high complexity datasets and shown scaffolding performance. The results are updated in Tables 1 and 4.*

3. It is not clear to me if the developed strategy depends from some free parameters (but it seems so looking at the webpage <https://github.com/marbl/MetaCarvel>). Please discuss in the text how they may influence the results.

*We have added text to the discussion section describing the impact of these parameters on the final output.*

4. A key point in the performance of the proposed tool is represented by the sequencing depth. I think you should evaluate this aspect in the manuscript in a more systematic way.

*We have performed the sequencing depth analysis by downsampling Singer et al dataset. The results are presented in Table 4.*

5. What in terms of computational load of the proposed tools? Please report some empirical evaluations.

*We have added the runtime and memory requirements for MetaCarvel for a couple of scaffolding experiments to the manuscript.*

**Second round of review**

**Reviewer 2**

The authors have improved their manuscript in this revised version and answered positively to my previous comments. I do not have additional suggestions.
